# Epidemiological survey to establish thresholds for influenza among children in satellite cities of Tokyo, Japan, 2014–2018

**DOI:** 10.5365/wpsar.2022.13.3.911

**Published:** 2022-08-25

**Authors:** Ayako Matsuda, Kei Asayama, Taku Obara, Naoto Yagi, Takayoshi Ohkubo

**Affiliations:** a Center for Public Health Informatics, National Institute of Public Health, Saitama, Japan.; b Department of Hygiene and Public Health, Teikyo University School of Medicine, Tokyo, Japan.; c Tohoku Institute for Management of Blood Pressure, Sendai, Japan.; d Studies Coordinating Centre, Research Unit, Hypertension and Cardiovascular Epidemiology, KU Leuven Department of Cardiovascular Sciences, University of Leuven, Leuven, Belgium.; e Tohoku Medical Megabank Organization, Tohoku University, Sendai, Japan.; f Warabi-Toda Medical Association, Toda, Japan.

## Abstract

**Objective:**

We described the characteristics of children reported as having influenza across five consecutive influenza seasons and investigated the usefulness of setting influenza thresholds in two satellite cities of Tokyo, Japan.

**Methods:**

An annual survey was conducted among parents of children at preschools (kindergartens and nursery schools), elementary schools and junior high schools in Toda and Warabi cities, Saitama prefecture, at the end of the 2014–2018 influenza seasons. Using the World Health Organization method, we established seasonal, high and alert thresholds.

**Results:**

There were 64 586 children included in the analysis. Over the five seasons, between 19.1% and 22% of children annually were reported as having tested positive for influenza. Influenza type A was reported as the dominant type, although type B was also reported in more than 40% of cases in the 2015 and 2017 seasons. The median period of the seasonal peak was 3 weeks in mid-January, regardless of school level. Of the five surveyed seasons, the high threshold was reached in 2014 and 2018, with no season exceeding the alert threshold.

**Discussion:**

This study provides insights into the circulation of influenza in children in the study areas of Toda and Warabi, Japan, from 2014 to 2018. Although we were able to utilize these annual surveys to calculate influenza thresholds from five consecutive seasons, the prospective usefulness of these thresholds is limited as the survey is conducted at the end of the influenza season.

The World Health Organization (WHO) estimates that annual epidemics of influenza cause 3–5 million cases of severe illness worldwide. (*1*) The epidemiology of influenza changes markedly each year and varies in different locations. (*2*) In general, approximately 80% of influenza cases are caused by influenza type A, whereas influenza type B accounts for approximately 20% of total global cases. (*3*)

Schoolchildren are the primary vulnerable population for influenza because they have the highest rates of influenza transmission and infection among infected populations. (*4*) In the Asia-Pacific region, influenza type B appeared to cause more illness in children between the ages of 1–10 years than in other age groups. (*5*) Although influenza surveillance data have been reported in various forms for populations across Japan, (*6*-*8*) few studies have investigated seasonal influenza among schoolchildren in and around Tokyo, the capital city of Japan and the most populous metropolitan area of the country.

Owing to the various thresholds for influenza epidemics, (*9*-*12*) WHO has proposed global standards for the collection, reporting and analysis of seasonal influenza epidemiological surveillance data. (*9*) The WHO further recommends obtaining average epidemic curves plus seasonal and alert thresholds as established tools to help control annual influenza epidemics. (*9*) The thresholds using the WHO methods are simple to implement and can be adapted easily for any influenza surveillance system with adequate historical data. (*13*) In some countries, the WHO method is used to inform key decision-makers for influenza outbreak management and public health action. (*14*-*16*)

We conducted a survey of children (from preschool to junior high school) during five consecutive influenza seasons in two satellite cities of Tokyo, Japan. Using these data, we described the characteristics of circulating influenza and investigated the usefulness of establishing thresholds for the influenza epidemic with the WHO method. To our knowledge, this is one of the first documented assessments using the WHO method to set thresholds for children in cities near Tokyo, Japan, based on survey data.

## Methods

### Study area

The study area comprised two cities, Toda and Warabi, which are located in Saitama prefecture to the north of Tokyo. The study region was 23.3 km^2^ (Toda: 18.2 km^2^; Warabi: 5.1 km^2^) and had a population of 208 410 (Toda: 136 150; Warabi: 72 260), including a population of 28 056 aged 0–14 years (Toda: 20 252; Warabi: 7804) according to the 2015 census. (*17*)

### Study procedure

Throughout five consecutive influenza seasons, from 2014 to 2018 (ending March 2019), an annual survey was conducted among parents of children who were attending preschool (kindergarten or nursery school, 0–6 years old), elementary school (7–12 years old) or junior high school (13–15 years old) in the Toda and Warabi regions. A questionnaire was mailed to parents asking for the following information regarding their children: school level, sex, siblings, underlying medical condition, vaccination status, and incidence of influenza infection, influenza type and date of illness (**Supplementary Table 1**

Click here for additional data file.).

In clinical practice in Japan, the influenza type (type A or B) is typically diagnosed by the children’s local physician or an emergency outpatient health-care provider, who administers an influenza antigen rapid test covered by health insurance. The survey was conducted every June, and the responses pertained to the preceding season. Completed questionnaires were collected by schoolteachers.

### Statistical analysis

We determined the number of children, percentage of influenza cases by type and week for each influenza season, and the seasonal, high and alert thresholds for influenza. The data were also analysed by school level (preschool, elementary school and junior high school; in Japan, there is no system for these schoolchildren to repeat the school year). Comparisons between those with and without reported influenza infection were compared using the χ^2^ test.

Each influenza season was defined as beginning in October and ending in March of the following year; for example, the 2014 season began in October 2014 and ended in March 2015. The epidemic peak was defined for each influenza type as the week with the highest number of reported influenza cases.

Data were extracted from the pooled survey responses of the five consecutive influenza seasons. In accordance with the WHO protocol, (*9*) we calculated the average and upper limit of the 90% confidence interval (CI) curves and the seasonal, high, and alert thresholds based on the number of children reported as having influenza each week throughout the five seasons. The average curves denoted the peak weekly mean, and the 90% upper curve was for the upper limits of the 90% CI of the peak weekly mean. (*9*, *13*) For these curves, the WHO protocol suggests using the normal distribution to assign thresholds based on the mean and standard deviation of the aligned data for weekly counts. (*9*) The seasonal threshold was defined as the annual median amplitude of the number of children reported with influenza per week throughout the study period. Therefore, half of the study weeks are necessarily above the seasonal threshold, and these correspond to the seasonality in the influenza epidemic (e.g. from week 40 of 2014 to week 13 of 2015).

The high threshold was defined as the number of children with influenza higher than the average peak for each of the five seasons, that is, the peak number of children with influenza of the average epidemic curves. (*15*) Theoretically, we can expect that seasonal peaks can be higher than the high threshold in two or three of the five seasons, whereas the seasonal peaks will be lower in other seasons. Finally, we defined the alert threshold as being higher than the upper limits of the 90% CI of the high threshold as defined earlier. (*9*, *13*, *15*) The data for the total number of children studied and for each school level from week 40 of 2014 to week 13 of 2019 were plotted against the calculated seasonal, high and alert thresholds. We analysed the data using Stata version 16.0 (Stata Corp., College Station, TX, United States of America).

## Results

**Figure 1:**
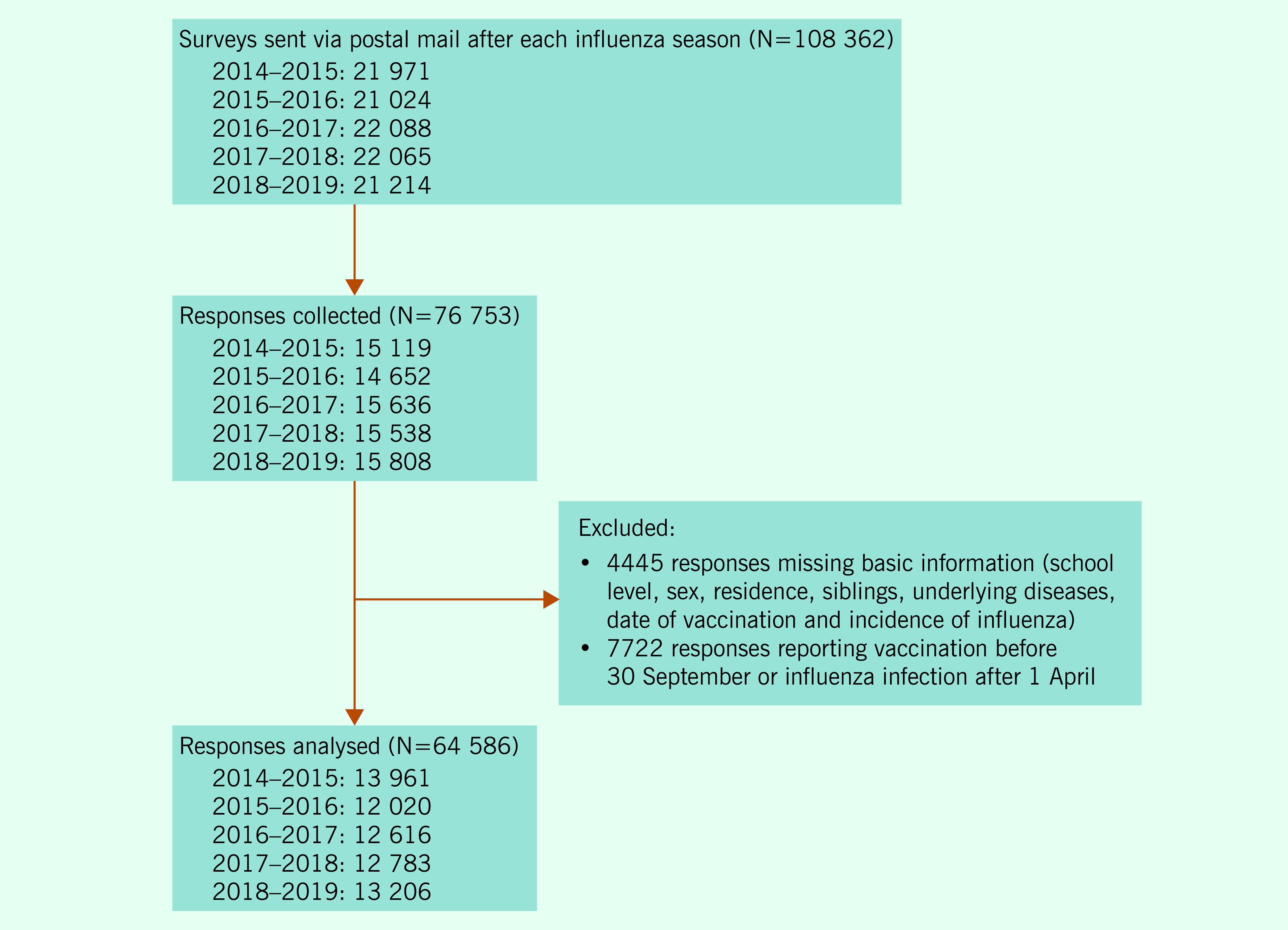
Selection of the study population for consecutive annual surveys of schoolchildren in Toda and Warabi, Japan, during the 2014–2018 influenza seasons

A total of 76 753 responses (response rate 70.8%) were collected from the 108 362 surveys sent to parents of children attending preschool, elementary school or junior high school during the 2014–2018 seasons. We excluded responses that did not include basic information (*n* = 4445) and those that reported influenza vaccination before 30 September or influenza infection after 1 April for each season (*n* = 7722). (*18*) This analysis, therefore, consisted of 64 586 responses (**Fig. 1**).

[**insert** Figure 1]

Of the included children, 49.6% were male, 78.6% had siblings and 8.3% had an underlying medical condition (**Table 1**). Among preschoolchildren, having siblings and the presence of underlying medical conditions were associated with influenza infection (*P* < 0.001). In elementary schoolchildren, sex and having siblings were associated with influenza infection (*P* < 0.001 and = 0.026, respectively). Conversely, sex, having siblings and the presence of underlying medical conditions were not associated with influenza infection in junior high schoolchildren (*P* = 0.103, 0.713 and 0.405, respectively) (**Table 1**).

**Table 1:** Comparison of characteristics of schoolchildren included in consecutive annual influenza surveys in Toda and Warabi, Japan, during the 2014–2018 influenza seasons

Characteristic	Total	Influenza infection reported, *n*(%)		*P* ^a^
		Yes	No	
	*n* = 64 586	*n* = 13 754	*n* = 50 832	
School				
Preschool (0–6 years)	17 260	3262 (18.9)	13 998 (81.1)	< 0.001
Elementary school (7–12 years)	34 966	8186 (23.4)	26 780 (76.6)	
Junior high school (13–15 years)	12 360	2306 (18.7)	10 054 (81.3)	
Sex				
Male	32 039	7037 (51.2)	25 002 (49.2)	< 0.001
Female	32 547	6717 (48.8)	25 830 (50.8)	
Siblings (yes)	50 756	10 888 (79.2)	39 868 (78.4)	0.064
Underlying medical condition (yes)	5347	1220 (8.9)	4127 (8.1)	0.005
Preschoolchildren (0–6 years)				
Sex (male)	8611	1663 (51.0)	6948 (49.6)	0.166
Siblings (yes)	12 020	2386 (73.2)	9634 (68.8)	< 0.001
Underlying medical condition (yes)	1397	319 (9.8)	1078 (7.7)	< 0.001
Elementary schoolchildren (7–12 years)				
Sex (male)	17 378	4210 (51.4)	13 168 (49.2)	< 0.001
Siblings (yes)	28 392	6578 (80.4)	21 814 (81.5)	0.026
Underlying medical condition (yes)	2987	731 (8.9)	2256 (8.4)	0.152
Junior high schoolchildren (13–15 years)				
Sex (male)	6050	1164 (50.5)	4886 (48.6)	0.103
Siblings (yes)	10 344	1924 (83.4)	8420 (83.8)	0.713
Underlying medical condition (yes)	963	170 (7.4)	793 (7.9)	0.405

^a^ Data from influenza infection (Yes) and influenza infection (No) were compared using the χ^2^ test.

### Children with influenza and their distribution by influenza type

The total number of children who were reported to have been infected with influenza was 13 754 (21.3% of analysed responses). With respect to the dominant influenza type in each season, type A dominated in 2014, 2016 and 2018, while type B dominated in 2017 and the two were nearly equal in 2015. These patterns mostly held when divided by school level (**Table 2**).

**Table 2:** Number of children reported with influenza and week of the epidemic peak in consecutive annual surveys of schoolchildren in Toda and Warabi, Japan, during the 2014–2018 influenza seasons

Season	Cases (%)/total no. of children	Influenza type reported (%)			Week of epidemic peaks		
		Type A	Type B	Unknown	All	Type A	Type B
All children							
2014	2793 (20.0)/13 961	80.2	11.6	8.2	51	51	51
2015	2594 (21.6)/12 020	45.7	43.7	10.6	6	5	9
2016	2770 (22.0)/12 616	71.6	17.9	10.5	51	51	12
2017	3070 (24.0)/12 783	28.7	45.9	25.4	3	3	5
2018	2527 (19.1)/13 206	84.3	6.6	9.1	3	3	3
Total	13 754 (21.3)/64 586	61.2	25.7	13.1	N/A	N/A	N/A
Preschool (0–6 years)							
2014	659 (17.3)/3 809	79.2	11.1	9.7	2	2	7
2015	614 (18.5)/3 321	48.4	41.0	10.6	5	5	7
2016	688 (20.5)/3 348	70.8	17.3	11.9	3	5	12
2017	701 (20.2)/3 472	37.8	42.9	19.3	5	2	5
2018	600 (18.1)/3 310	85.3	7.0	7.7	2	2	3
Subtotal	3262 (18.9)/17 260	63.9	24.1	12.0	N/A	N/A	N/A
Elementary school (7–12 years)							
2014	1567 (21.6)/7 269	80.6	11.5	7.9	51	51	51
2015	1672 (25.9)/6 445	45.9	42.6	11.5	6	6	9
2016	1503 (22.1)/6 793	71.1	19.0	9.8	3	51	12
2017	1905 (28.0)/6 807	28.7	49.0	22.3	3	51	5
2018	1539 (20.1)/7 652	83.6	6.4	10.0	3	3	3
Subtotal	8186 (23.4)/34 966	60.2	27.0	12.8	N/A	N/A	N/A
Junior high school (13–15 years)							
2014	567 (19.7)/2 883	80.4	12.5	7.1	51	51	51
2015	308 (13.7)/2 254	39.3	55.2	5.5	7	5	10
2016	579 (23.4)/2 475	73.6	15.7	10.7	51	51	12
2017	464 (18.5)/2 504	15.1	37.5	47.4	3	51	52
2018	388 (17.3)/2 244	85.6	6.7	7.7	3	3	3
Subtotal	2306 (18.7)/12 360	60.9	23.1	16.0	N/A	N/A	N/A

N/A: not available.

The influenza season begins in October and ends in March of the following year; for example, the 2014 season was from October 2014 to March 2015.

### Week of epidemic peak by influenza type

The epidemic peaks occurred earlier in 2014 and 2016 (week 51) than in 2015 (week 6), 2017 (week 3) and 2019 (week 3) (**Table 2**). The epidemic peaks of influenza type B occurred later than type A in 2015, 2016 and 2017. By school level, the epidemic peaks in preschool occurred later than the other levels in 2014, 2016 and 2017 (**Table 2**).

### Curves and thresholds by the WHO method

**Figure 2:**
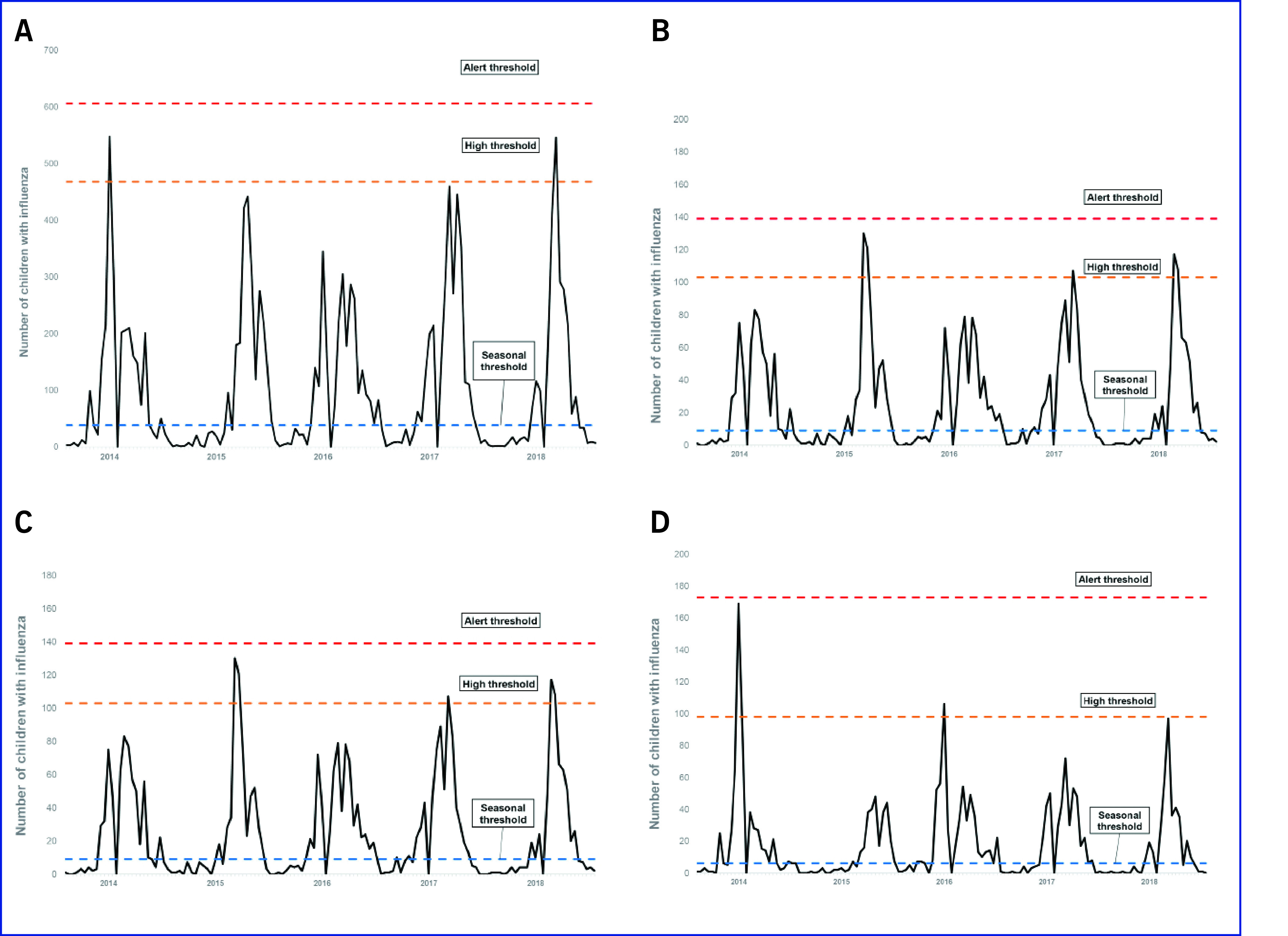
Number of reported influenza cases from consecutive annual surveys of schoolchildren in Toda and Warabi, Japan, during the 2014–2018 influenza seasons plotted against the calculated WHO thresholds (A) overall, and for (B) preschool, (C) elementary and (D) junior high school children

The start of the influenza season was between weeks 43 and 1 (late October and early January), and the end of the season was between weeks 8 and 13 (late February and late March). The median peak in the number of children with influenza was similar to the corresponding mean peak (**Table 3**). The median week of the peak was week 3 (mid-January; **Table 3**). The plotted curve of the number of children with influenza crossed the seasonal threshold multiple times over the five seasons. The peak in seasonal influenza activity in 2015, 2016 and 2017 did not reach the high threshold (**Fig. 2A**).

[**insert** Figure 2]

**Table 3:** Epidemic curve characteristics and thresholds in consecutive annual surveys of schoolchildren in Toda and Warabi, Japan, during the 2014–2018 influenza seasons

-	Total	School level		
		Preschool	Elementary school	Junior high school
Median week of peak	3	3	3	3
Median peak in influenza cases	460	107	299	97
Mean peak in influenza cases	468.2	103.2	279.4	98.4
Standard deviation	84.2	21.9	63.9	45.5
Upper 90% confidence interval	606.7	139.2	384.5	173.2
Upper 95% confidence interval	633.2	146.1	404.6	187.6
Threshold level	–	–	–	–
Seasonal threshold	38	9	21	6
High threshold	468	103	279	98
Alert threshold	606	139	384	173

The influenza season begins in October and ends in March of the following year; for example, the 2014 season was from October 2014 to March 2015.

The peak in seasonal influenza activity varied when the children with influenza were stratified by school level (**Fig. 2B–D**). In none of the five seasons did the plotted curve of the prevalence of children with influenza cross the alert threshold. The results were almost confirmatory when classified by school level, except for junior high school during the 2014 season, where the number of children with influenza was close to the alert threshold (**Fig. 2D**).

## Discussion

We present data on the circulation of influenza in children who were attending preschool, elementary school or junior high school in Toda and Warabi, Japan, during five consecutive influenza seasons from 2014 to 2018. Over the five seasons, between 19.1% and 22% of children annually were reported as having tested positive for influenza. Over the whole period, there was a higher proportion of elementary schoolchildren reporting influenza infection (23.4%) compared to preschool and junior high schoolchildren (18.9% and 18.7%, respectively). Having siblings was associated with reported cases of influenza in preschool and elementary schoolchildren. Moreover, we successfully established seasonal, high and alert thresholds based on survey data from five consecutive seasons of influenza using the WHO method.

In Japan, the Ministry of Health, Labour and Welfare, in collaboration with the National Institute of Infectious Diseases (NIID), provides a weekly influenza outbreak report. (*19*) This report is based on a school survey in which the absence of children and temporary closure of schools are recorded. The total number of temporary school closures was highest in 2017, which supports our finding that the highest number of reported influenza cases also occurred in 2017. Our survey differed from this national report (*19*) for junior high schoolchildren, as the highest number of influenza cases was reported in 2016 for this group.

In our survey, approximately 40% of influenza cases in 2015 and 2017 were type B. These results are similar to those reported in NIID’s influenza outbreak summaries for each season, (*19*) although their proportion of type B reported among children in junior high school was higher, at > 50% in 2015. Characteristics of outbreaks can differ by region, even within a single country, warranting local-level surveys.

In the national report, (*19*) the peak week for temporary school closures occurred in weeks 4, 7, 4, 5 and 4 in the 2014, 2015, 2016, 2017 and 2018 seasons, respectively. The week of the influenza epidemic peak in our survey occurred consistently earlier than that in the national report, although the overall tendency was similar. This may be because the national report used the dates of school absence due to influenza, (*19*) whereas our survey showed the week with the highest number of detected influenza cases which is likely to precede the week of temporary school closures. There may also be regional characteristics that contribute to differences in the national patterns.

The increase in reported influenza type B cases in the national data occurred later than type A in our survey. The epidemic order is in accordance with that observed in other influenza seasons in the northern hemisphere. (*20*) Understanding the geographical and temporal patterns of seasonal influenza could help strengthen influenza surveillance for the early detection of epidemics. (*21*) As Mosnier et al. reported, (*22*) timely data on the circulation of influenza collected by influenza surveillance systems are essential for optimizing influenza prevention and control strategies. (*21*, *22*)

In accordance with the WHO method, we developed three thresholds (seasonal, high and alert thresholds) for children at each school level in two satellite cities of Tokyo, based on survey data from the same region. The WHO method is a simple protocol to establish influenza thresholds. Epidemic peaks for each season occurred at week 51 or later, particularly at week 2 or later among preschoolchildren. Two of the five seasons, 2014 and 2018, reached the high threshold; none of the seasons reached the alert threshold.

The data used in this study were not collected in a near real-time manner and are not surveillance data for which the threshold calculations are best suited. Therefore, the calculated thresholds cannot be used to establish an outbreak warning system; they can only be used to assess an influenza season after its completion. This is in contrast to the influenza surveillance system in Japan which provides alerts throughout the influenza season when the reported number of cases exceeds the threshold in any given week. (*23*) However, the annual survey is cost-effective and feasible and can provide a retrospective assessment of an influenza season in a subgroup of the population. Furthermore, the established thresholds can be used to guide public health decision-making and risk communication for children, for example by planning national and municipal budgets and long-term staffing as well as preparing for periods and intensive education for children when epidemics are expected. The thresholds can also be helpful in establishing an early warning system for influenza epidemics customized to each region when a near real-time report such as the aforementioned NIID report in Japan (*19*) is feasible and can facilitate collaboration.

Our study has several limitations. First, preschool-aged children who were not attending kindergarten or nursery school, and children who were attending school out of town, were excluded from the analysis. In the study area (Toda and Warabi), the total number of children aged £15 years was 27 562 according to the 2015 census. As only 60% of mailed surveys were returned and qualified for analysis, we cannot guarantee that the present findings accurately represent the epidemiology of children in the general population. Second, as the questionnaires were answered by the parents of the targeted children, influenza diagnosis was based on self-reporting. Detailed medical information was not requested, so the proportion reported with influenza might not be accurate. Third, not all participants completed all five surveys that were conducted for this report. As the last survey was completed in March 2019, the data were not affected by the COVID-19 pandemic and related confounding circumstances. Whether the current estimates regarding the influenza epidemic will be applicable after the COVID-19 pandemic has subsided remains unknown; this is the same issue for the epidemiology of most infectious diseases.

This study provides insights into the circulation of influenza in children in the study areas of Toda and Warabi. The calculated thresholds provide some assessment of the influenza seasons from 2014 to 2018 in this group and the epidemic curve information may help prepare for the health care of children as the influenza season starts. If this survey data could be collected routinely during the influenza season, then the thresholds may contribute to an early warning system; currently, they can only be used to assess influenza seasons after they have occurred. Our findings based on an influenza survey of children are useful for general practitioners, health policy-makers and disease control planners who are concerned with the prevention and control of influenza in this local area.
