# Supplementary material for: Epidemiological survey to establish thresholds for influenza among children in satellite cities of Tokyo, Japan, 2014–2018
Source: Western Pac Surveill Response J. 2022 Aug 25;13(3):1–9. doi: 10.5365/wpsar.2022.13.3.911 (PMC9671205; doi:10.5365/wpsar.2022.13.3.911)
Supplement: Supplementary file 1 [file wpsar-13-911-s001.pdf]

Supplementary Table 1. **Influenza survey (2018–2019 season)**

**Q1— Questions about your child.**		
Q1-1 Sex: Male/Female		
Q1-2 School		
1: Nursery school (age: 0–3 years)		2: Kindergarten or nursery school (age: 4 years)
3: Kindergarten or nursery school (age: 5 years)		4: Kindergarten or nursery school (age: 6 years)
5: Elementary school (age: 7–12 years), grade [ ]		
6: Junior high school (age: 13–15 years), grade [ ]		
Q1-3 Birthday (year/month): ______/____		
Q1-4 Sibling(s): No/Yes		
Q1-5 Underlying disease: No/Yes		
If yes, (a) Asthma, (b) Epilepsy, (c) Other: __________		
Q1-6 Did your child live in Toda or Warabi city during the season? Yes/other place: ______		
Q1-7 What is the method of transportation used by your child for going to school (walking, by bus, etc.)? ____________________		
**Q2— Questions about infection prevention.**		
Q2-1 Frequency of hand washing		
1: Never	2: Somewhat	3: Frequently
Q2-2 Frequency of mask-wearing		
1: Never	2: Somewhat	3: Frequently
**Q3— Questions about influenza (flu) vaccination.**		
Q3-1 Vaccination status		
1: None	2: Vaccinated once during the season	3: Vaccinated twice during the season
Q3-2 Vaccination status with date, if done		
First time (year/month/day): 201__/____/____		
Second time (year/month/day): 201__/____/____		
**Q4— Questions about influenza infection.**		
Q4-1 Did your child have influenza?		No/Yes
Q4-2 If yes, infection date and type of influenza during the season		
First infection (year/month/day): 201__/____/____		Type of influenza: A/B/Unknown
Second infection (year/month/day): 201__/____/____		Type of influenza: A/B/Unknown
Q4-3 Was your child tested for influenza?		No/Yes/Unknown
Q4-4 Did your child receive any treatment for influenza? (Select all that apply)		
1. Medicine: Oseltamivir (Tamiflu®)		2. Medicine: Baloxavir (Xofluza®)
3. Spray: Zanamivir (Relenza®)		4. Spray: Laninamivir (Inavirl®)
5. Intravenous: Peramivir (Rapiacta®)		6. Other: ____________
7. Unknown		
